# Evaluation of meningeal lymphatic vessels with delayed contrast enhanced vessel-wall MRI in cerebral infarction

**DOI:** 10.1007/s11604-025-01908-0

**Published:** 2025-11-10

**Authors:** Masaki Umehana, Yasutaka Fushimi, Sachi Okuchi, Sayo Otani, Takayuki Yamamoto, Akihiko Sakata, Satoshi Nakajima, Satoshi Ikeda, Shuichi Ito, John Grinstead, Sinyeob Ahn, Katsuhiko Mitsumoto, Kentaro Ueno, Chia-Hung Wu, Satoshi Miura, Takakuni Maki, Hideo Chihara, Takeshi Funaki, Yoshiki Arakawa, Yuji Nakamoto

**Affiliations:** 1 Department of Diagnostic Imaging and Nuclear Medicine, Kyoto University Graduate School of Medicine, 54 Shogoin Kawahara-cho, Sakyo-ku, Kyoto, 606-8507 Japan; 2 Siemens Healthineers, Portland, OR USA; 3 Siemens Healthineers, San Francisco, CA USA; 4 Department of Clinical Radiology Service, Kyoto University Hospital, Kyoto, Japan; 5 Department of Biomedical Statistics and Bioinformatics, Graduate School of Medicine, Kyoto University, Kyoto, Japan; 6 Department of Radiology, Taipei Veterans General Hospital, Taipei, Taiwan; 7 Department of Neurology, Graduate School of Medicine, Kyoto University, Kyoto, Japan; 8 Department of Neurosurgery, Graduate School of Medicine, Kyoto University, Kyoto, Japan

**Keywords:** Meningeal lymphatic vessels, Intramural periarterial drainage, Glymphatic system, Vessel-wall MRI, Stroke

## Abstract

**Purpose:**

Observation of the clearance of gadolinium-based contrast agents (GBCA) in patients with cerebral infarction and Moyamoya disease (MMD) using vessel-wall MRI (VW-MRI).

**Methods:**

This prospective study included 11 patients with recent cerebrovascular disorder and 6 patients with MMD who were scheduled for VW-MRI. All participants underwent whole brain delay alternating with nutation for tailored excitation-prepared T1-weighted variable flip angle turbo spin echo (DANTE T1-SPACE) imaging before, immediately after, and 1–6 h after gadolinium injection. Additionally, a phantom experiment was conducted to assess the signal intensity according to T1 values using DANTE T1-SPACE and 3D real inversion recovery sequences.

**Results:**

All five large infarction cases (≥ 5 cm) had delayed meningeal enhancement on the ipsilateral side, while none of the remaining six small infarction cases (< 5 cm) exhibited delayed meningeal enhancement on MRI. More than half of the entire cases had delayed white matter enhancement adjacent to the infarction. No MMD cases showed delayed enhancement outside postoperative regions. In the phantom experiment, DANTE T1-SPACE (TR = 700–1300 ms) demonstrated stable signal intensity across a T1 range of approximately 500–1000 ms. This T1 range corresponds to signal intensity ≈ 200–600 typically observed in regions showing delayed enhancement in clinical stroke and MMD cases.

**Conclusions:**

Delayed meningeal enhancement observed in patients with large cerebral infarctions likely reflects alterations in brain clearance pathways. The absence of such enhancement in MMD supports that these enhancements are not attributable to collateral circulation. Phantom validation confirmed that the imaging parameters used in the DANTE T1-SPACE sequence were sufficient to detect subtle contrast enhancement in the clinically relevant T1 range.

## Introduction

Delayed-phase contrast-enhanced (CE) MRI, typically acquired 1–6 h after gadolinium-based contrast agents (GBCA) administration, enables the visualization of slow-moving or retained contrast agents in interstitial fluid (ISF) clearance of the brain. Unlike conventional early-phase imaging, which captures arterial or venous enhancement or blood-brain barrier (BBB) leakage within minutes of injection, delayed imaging has been reported to be useful in various clinical settings, including the detection of brain metastases [1], differentiation between tumor recurrence and radiation necrosis [2, 3], assessment of treatment response in glioblastoma [4, 5], identification of active lesions in multiple sclerosis [6], and evaluation of endolymphatic hydrops in Ménière’s disease [7, 8].

More recently, delayed-phase imaging has also been applied to investigate ISF clearance pathways in the central nervous system (CNS), particularly the glymphatic system and meningeal lymphatic vessels (MLVs). The glymphatic system is a perivascular fluid transport pathway that facilitates the clearance of interstitial solutes from the brain, including metabolic waste and proteins such as amyloid-β [9]. Impaired glymphatic flow has been implicated in aging and various neurological disorders [10]. Non-invasive visualization of glymphatic activity has been attempted using intrathecal or intravenous contrast-enhanced MRI, where delayed accumulation of contrast in perivascular spaces (PVS) serves as an indirect marker of glymphatic transport [11].

In parallel, MLVs—distinct lymphatic structures located within the dura mater—have been visualized using high-resolution CE MRI, such as T1 black blood images, FLAIR. MLVs are most commonly detected adjacent to the superior sagittal sinus (SSS) and transverse sinuses, with signal enhancement observed several hours after intravenous contrast administration. MLVs are considered downstream drainage pathways of the glymphatic system, facilitating the removal of interstitial solutes from the brain. The ability to visualize these structures in vivo using delayed CE MRI has provided a new window into brain-wide waste clearance mechanisms and their alterations in disease states.

This study was motivated following an incidental observation in a stroke patient who underwent brain MRI five hours after GBCA administration, which revealed unexpected enhancement along the dura mater, pia mater, and cerebrospinal fluid (CSF) space on tailored excitation-prepared T1-weighted variable flip angle turbo spin echo (DANTE T1-SPACE). As in our case, vessel wall MRI (VW-MRI) has also been used to evaluate MLVs in humans [12]. In addition, VW-MRI using DANTE pulses also allows efficient suppression of the signals of moving protons associated with arteries, veins, and CSF [13], which might make it suitable for evaluation of slow lymphatic vessels [14, 15]. The spatial distribution and delayed timing of this enhancement raised the possibility that it might reflect clearance of contrast agent through MLVs.

The purpose of this study was to prospectively observe the chronological changes in contrast agent distribution within the dura mater, subarachnoid space, and perivascular regions—anatomical compartments potentially involved in glymphatic and meningeal lymphatic drainage. To further distinguish this enhancement from vascular contributions, especially from collateral circulation, we additionally included an exploratory cohort of patients with moyamoya disease (MMD) who underwent CE VW-MRI for evaluation of disease activity [16]. Given that MMD is known for extensive leptomeningeal collaterals and post-surgical anatomical changes, this comparison allowed us to assess whether the delayed enhancement observed in stroke patients could be attributed to lymphatic outflow rather than abnormal or slow vascular flow.

## Materials and methods

### Participants

This prospective study was performed in accordance with the Declaration of Helsinki and was approved by the local institutional review board (R3963). Written informed consent was obtained from all participants.

This study included consecutive inpatients managed by T.M., a stroke specialist at our institute, as the attending physician between September 2023 and July 2024. The inclusion criteria were as follows: (a) patients affected by recent cerebrovascular disorders (CVD) scheduled for CE MRI, including VW-MRI, (b) no history of severe allergic reactions or anaphylaxis to GBCA, (c) absence of severe renal impairment, and (d) ability to provide informed consent. The exclusion criteria were insufficient image quality due to motion artifacts.

Additionally, as an exploratory cohort, to evaluate whether meningeal enhancement could be attributed to MLVs rather than collateral vasculatures, we included 6 patients with MMD, a condition characterized by well-developed leptomeningeal collaterals.

### Image acquisition

MRI was performed using a 3T scanner (MAGNETOM Prisma; Siemens Healthineers, Erlangen, Germany) with a 64-channel head/neck coil. In this study, whole brain DANTE T1-SPACE images were obtained as vessel wall MRI with the following parameters: sagittal 3D acquisition, TR/TE, 1000/11 ms; field of view (FOV) 210 × 240 mm^2^; matrix, 378 × 432; slice thickness, 0.56 mm; 256 contiguous slices; acceleration with controlled aliasing in parallel imaging results in higher acceleration (CAIPIRINHA), 4×; and acquisition time, 6 min 45 s. Parameters for the DANTE pulse were as follows: flip angle, 10°; radiofrequency (RF) duration, 0.08 ms; number of pulses, 148; total pulse duration, 167.24 ms; and spoiler gradient area, 18 mT/m×ms. DANTE T1-SPACE images were obtained before injection, immediately after GBCA (gadobutrol, 0.1 mmol/kg body weight, Bayer Pharmaceuticals, Berlin, Germany) injection, and 1–6 h after injection (called as NC DANTE T1-SPACE, CE DANTE T1-SPACE, Delayed DANTE T1-SPACE, respectively). Whole brain 3D FLAIR imaging (TR/TE, 5000/388 ms; inversion time, 1800 ms; flip angle, 120 degrees; sagittal 3D acquisition; FOV, 224 mm^2^; slice thickness, 1 mm; 288 contiguous slices; ) was also performed, although only during the delayed phase.

### Image analysis

Radiological data were anonymized, and postprocessing was performed by a board-certified neuroradiologist (M.U., with 8 years of experience in neuroradiology). Separately, three board-certified neuroradiologists (A.S., S.Ok., and S.Ot., with 17, 17 and 14 years of experience in neuroradiology, respectively) evaluated patients’ images for Gd leakage, defined as lesions with high signal intensities on Delayed DANTE T1-SPACE images. In MMD cohort, we also evaluated the dura mater, subdural space, subarachnoid space, and cortical veins within the postoperative area. The contrast enhancement was evaluated on a four-point visual scale (grade 0–3; grade 0: no enhancement, grade 1: uncertain enhancement, grade 2: slight enhancement, grade 3: moderate enhancement).

We classified the areas of contrast enhancement into four major categories based on the regions involved, as follows: Category A: white matter, pia mater, CSF space, and dura mater around the infarct; Category B: nearby cortical veins and venous sinuses (anterior, middle, and posterior parts of the SSS, and the sigmoid sinus): Category C: cranial nerves (CN) (olfactory nerve: CN I, optic nerve: CN II, oculomotor nerve: CN III, trigeminal nerve: CN V, facial nerve/vestibulocochlear nerve: CN VII/VIII); and Category D: miscellaneous regions (choroid plexus, anterior chamber, pineal gland, Eustachian tube, and auricular cartilage). For evaluation of veins, the evaluation targeted contrast enhancement in the surrounding regions.

Additionally, the contrast enhancement grade was summarized, with grade 0–1 considered as no enhancement and grade 2–3 considered as enhancement present.

The final results were based on the consensus of the three neuroradiologists if discrepancy existed.

### Statistical analysis

Interrater reliability for image quality scores measured independently by the three radiologists was evaluated using Fleiss’ kappa statistics. The calculated κ statistic was interpreted as follows: 0.20 or less, slight agreement; 0.21–0.40, fair agreement; 0.41–0.60, moderate agreement; 0.61–0.80, substantial agreement; and 0.81–1.00, almost perfect agreement.

All statistical analyses were performed with EZR (Saitama Medical Center, Jichi Medical University, Saitama, Japan) a graphical user interface for R (The R Foundation for Statistical Computing, Vienna, Austria) [17]. More precisely, it is a modified version of R commander designed to add statistical functions frequently used in biostatistics.

### Phantom experiment

We used the International Society for Magnetic Resonance in Medicine (ISMRM)/National Institute of Standards and Technology (NIST) MRI system phantom (CaliberMRI, Boulder, CO, USA) [18, 19]. The ISMRM/NIST MRI system phantom has multiple layers of sphere arrays that are designed to have a range of specific T1 values. The theoretical T1 values of the 14 spheres in the NIST phantom used for this experiment were as follows: T1-1: 1838 ms, T1-2: 1398 ms, T1-3: 998.3 ms, T1-4: 725.8 ms, T1-5: 509.1 ms, T1-6: 367 ms, T1-7: 258.7 ms, T1-8: 184.7 ms, T1-9: 130.8 ms, T1-10: 90.9 ms, T1-11: 64.2 ms, T1-12: 46.28 ms, T1-13: 32.65 ms, and T1-14: 22.95 ms. Spheres T1-11 to T1-14, located in the central region of the phantom, exhibit extremely short T1 values that are not physiologically encountered. Since this phantom study aimed to evaluate T1 values associated with post-contrast enhancement, the extremely short T1 values represented by these spheres were considered beyond the scope of our analysis. Therefore, only spheres T1-1 to T1-10 were included to ensure a physiologically relevant and technically reliable T1 assessment.

All scans were performed using a 3T scanner (MAGNETOM Skyra; Siemens Healthineers, Erlangen, Germany) with a 32-channel head coil. While maintaining their temperature at approximately 24.5 °C, the phantom images were then obtained using DANTE T1-SPACE with varying TR from 700 to 1300 ms at 100-ms intervals. The other parameters were set as follows: TE, 11 ms; flip angle, 120°; field of view (FOV), 180 × 180 mm^2^; matrix, 320 × 320; slice thickness, 0.56 mm; 256 contiguous slices; acceleration with controlled aliasing in parallel imaging results in higher acceleration (CAIPIRINHA), 4×; and acquisition time, 5 min 44 s. Parameters for the DANTE pulse were as follows: flip angle, 10°; radiofrequency (RF) duration, 0.08 ms; number of pulses, 148; total pulse duration, 167.24 ms; and spoiler gradient area, 18 mT/m×ms.

We additionally performed 3D inversion recovery with phase sensitive reconstruction (3D-real IR), which is commonly used for evaluation of Gd leakage, with varying inversion time from 1500 to 2700 ms at 150-ms intervals. The other imaging parameters of 3D-real IR were as follows: sagittal 3D acquisition, TR/TE, 15,130/547 ms; flip angle, 140°; FOV, 196 × 165 mm^2^; matrix, 384 × 326; slice thickness, 1 mm; 256 contiguous slices; acceleration with generalized autocalibrating partially parallel acquisition (GRAPPA), 3×; and acquisition time, 10 min 7 s.

ROI analysis was performed by measuring values of each sphere with a circular ROI of 100 mm^2^ which was manually drawn to exclude edge pixels.

## Results

### Participant characteristics

In total, 11 patients with cerebrovascular disorders (CVD) were included in this main study (mean age, 76.8 ± 8.3; age range, 55–85 years; 6 males, 5 females), with no exclusions (Table 1). All 11 patients had hypertension, four had diabetes mellitus, five had dyslipidemia, five had arrhythmia, and three were former smokers. Large infarcts (≥ 5 cm) were observed in five cases. The estimated time from infarct onset to MR imaging was 11.5 ± 4.5 days. The elapsed time after GBCA injection at the time of Delayed DANTE T1-SPACE acquisition was 4.8 ± 1.2 h.

**Table 1:** CVD patient demographics

	Sex	Age (years)	Interval (hour: min)	Infarct site	Hemorrhage	Size	HT	DM	DL	Af	Smoking history (years)	Estimated onset time (days ago)
Case 1	M	84	2:28	Rt. frontal lobe, Rt. insular cortex	+	3 cm, 1 cm	+	−	−	+	+, 42	Not determined (subacute)
Case 2	F	76	5:52	Lt. frontotemporal lobe	+	5 cm	+	+	−	−	−	18
Case 3	M	74	5:54	N/A (Lt. MCA M1 occlusion)	−	N/A	+	+	+	−	+, 30	1.6
Case 4	F	85	5:19	Rt. insular gyrus, Rt. frontal cortex	−	1 cm, 2 mm	+	−	+	+	−	11.2
Case 5	M	81	5:32	Rt. temporal occipital lobe	+	9 cm	+	−	−	+	−	12
Case 6	M	84	5:32	Rt. temporal lobe	+	9 cm	+	+	−	+	−	9
Case 7	F	77	4:45	Rt. cerebral hemisphere	+	13 cm	+	−	−	+	−	17
Case 8	F	55	2:44	Multiple infarctions due to Rt. ACA dissection	−	< 2 cm (multiple)	+	−	+	−	−	11
Case 9	M	76	5:35	Rt. corona radiata	−	< 2 cm	+	+	+	−	−	10
Case 10	F	83	5:15	Lt. internal capsule	−	1 cm	+	−	+	−	−	9
Case 11	M	70	3:33	Bil. occipital lobe, Rt. cerebellum	+	10 cm<	+	−	−	−	+, 30	16

Interval, interval between contrast injection and obtaining Delayed DANTE T1-SPACE images (hour: min); hemorrhage, hemorrhage at the infarct site; HT, hypertension; DM, diabetes mellitus; DL, dyslipidemia; Af, atrial fibrillation;

Duration, duration of smoking

In the MMD cohort, 6 patients were recruited (mean age, 41.8 ± 13.6 years; age range, 25–63 years; 3 males, 3 females) (Table 2). Five patients had undergone surgical revascularization, including superficial temporal artery to middle cerebral artery (STA–MCA) bypass (*n* = 5) and occipital artery to posterior cerebral artery (OA–PCA) bypass (*n* = 1). One patient had no history of prior surgery.

**Table 2:** MMD patient demographics

	Sex	Age	Interval	Affected MMD side, Suzuki staging	Surgery (days from surgery)	Infarction, size
Case 1	F	25	3:18	Right, N/A; left, 3	Left STA-MCA bypass (78)	N/A
Case 2	F	45	3:34	Right, N/A; left, 2	N/A	N/A
Case 3	M	48	3:12	Right, 3; left, 1	Left STA-MCA bypass (230); left OA-PCA bypass (125)	N/A
Case 4	M	63	3:07	Right, 3; left, 4	Left STA-MCA bypass (129)	Subacute, <1 cm
Case 5	M	42	3:03	Right, 4; left, 4	Right STA-MCA bypass (92)	N/A
Case 6	F	28	3:06	Right, 3; left, N/A	Right STA-MCA bypass (99)	N/A

Interval, interval between contrast injection and obtaining Delayed DANTE T1-SPACE images (hour: min);

STA-MCA, superficial temporal artery to middle cerebral artery; OA-PCA, occipital artery to posterior cerebral artery

### Image analysis

**Fig. 1:**
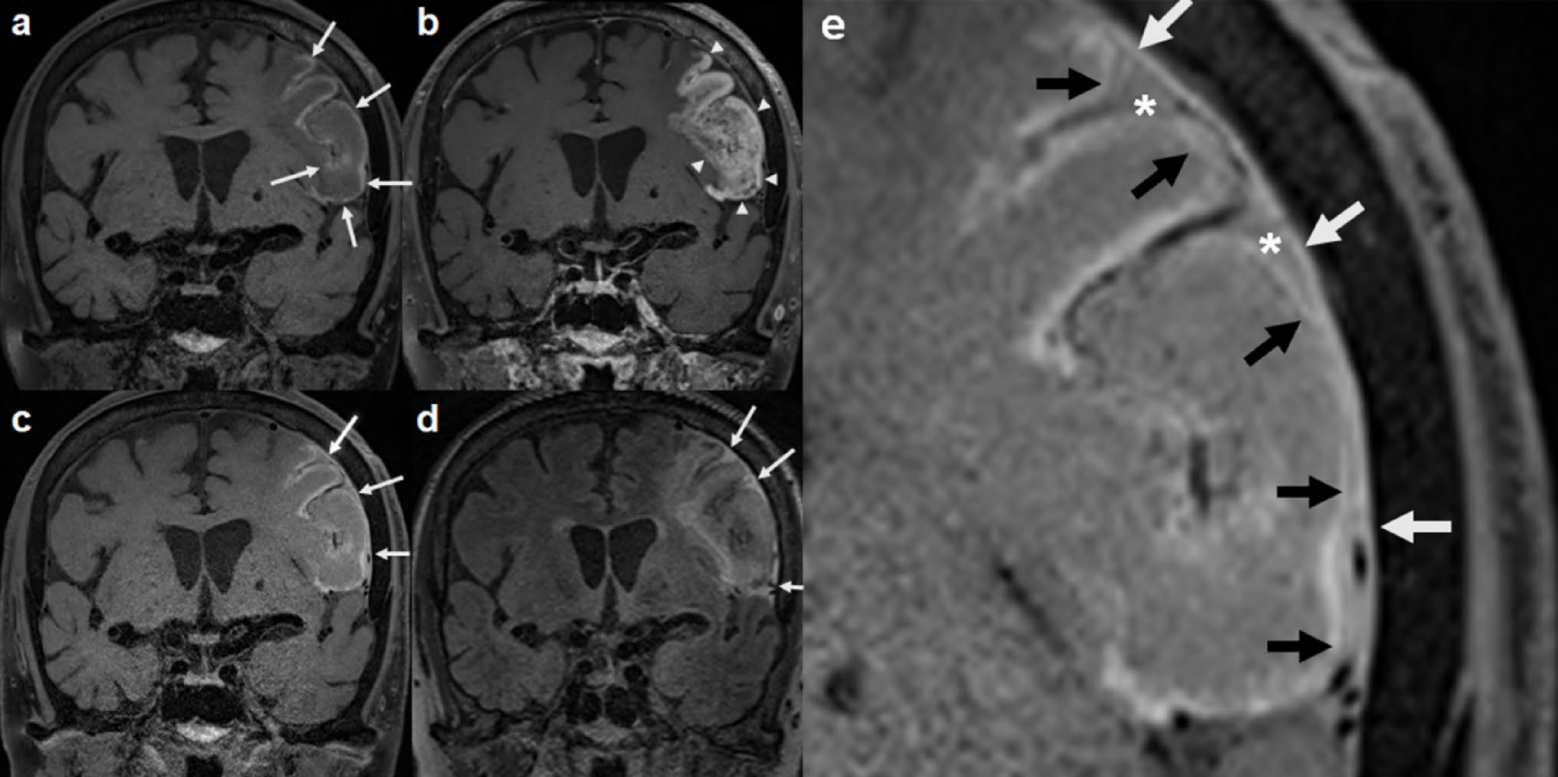
Representative images in a CVD patient (Case 2–CVD) with a large, subacute, left frontal lobe infarction. The NC DANTE T1-SPACE image **a** revealed regions of high signal intensity along the cortices, indicating hemorrhage or necrosis (arrows). The CE DANTE T1-SPACE image **b** acquired immediately after contrast injection showed contrast enhancement across the entire infarcted region (arrowheads). In the Delayed DANTE T1-SPACE image **c** obtained five hours after GBCA administration, the enhancement within the infarcted area had diminished, while new enhancement was observed along the dura mater adjacent to the infarct (arrows). Additionally, the subarachnoid space in that region exhibited contrast enhancement (c), which was obvious on FLAIR in the delayed phase (arrows) (**d**). Enlarged view of the Delayed DANTE T1-SPACE image **e** focusing on the infarcted region demonstrates enhancement along the dura mater (white arrows), pia mater (black arrows), and within the CSF space (asterisks)

**Fig. 2:**
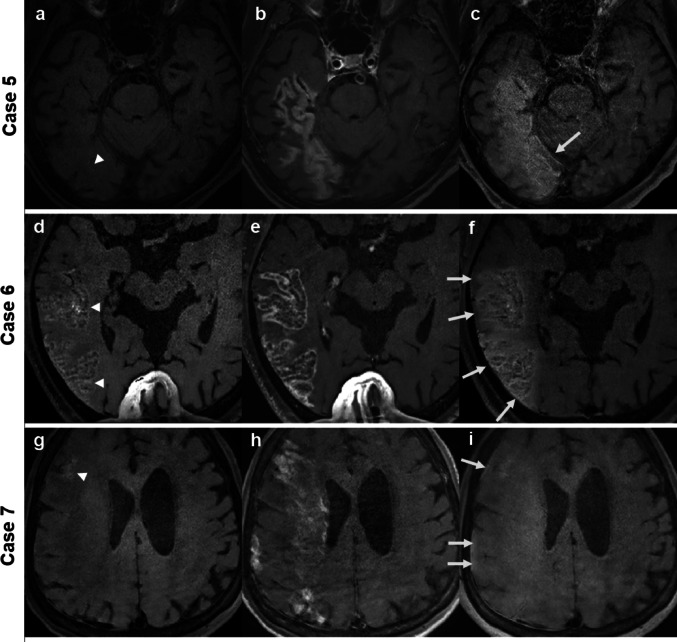
Representative images in CVD patients (Case 5–CVD, Case 6–CVD, and Case 7–CVD) with large subacute infarctions. NC DANTE T1-SPACE scans (**a**, **d**, **g**) revealed regions of high signal intensity (arrowheads), indicating hemorrhage or necrosis. CE DANTE T1-SPACE scans (**b**, **e**, **h**) showed contrast enhancement across the entire infarcted region. In the Delayed DANTE T1-SPACE scans (**c**, **f**, **i**), enhancement within the infarcted area had diminished, while new enhancement appeared along the dura mater adjacent to the infarct (arrows). Additionally, the subarachnoid space in that region exhibited contrast enhancement

**Fig. 3:**
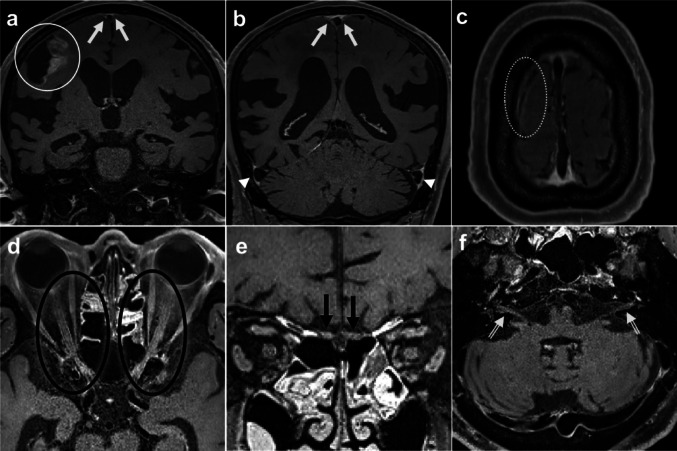
CNs on Delayed DANTE T1-SPACE images (Case 1–CVD, Case 3–CVD). In Case 1–CVD (**a**–**c**) with right-sided infarction (white circle), obvious enhancement around the SSS (white arrows), and sigmoid sinus (arrowheads) were scored grade 3. The enhancement around the bridging vein near the infarction was equivocal, and was scored as grade 1 (dotted circle). In Case 3–CVD (**d**–**f**) with severe left MCA stenosis, enhancement around CN II was scored grade 2 (black circle), that around CN I was scored grade 2 (black arrows), and that around CN VII & VIII was scored grade 2 (double arrows). CN, cranial nerves; I, olfactory nerve; II, optic nerves; VII, facial nerve; VIII, vestibulocochlear nerve; MCA, middle cerebral artery; SSS, superior sagittal sinus. CVD, cerebrovascular disorder

Images from representative CVD cases are shown in Figs. 1, 2, and 3. In cases with large infarctions (≥ 5 cm), NC DANTE T1-SPACE images revealed high signal intensity lesions, indicating hemorrhage or necrosis, CE DANTE T1-SPACE images acquired immediately after GBCA administration showed contrast enhancement across the entire infarcted regions, and Delayed DANTE T1-SPACE images showed diminution of the enhancing lesions within the infarcted area, while new enhancement appeared along the dura mater adjacent to the infarct. Additionally, the subarachnoid space in the same region exhibited contrast enhancement, which was also obvious on FLAIR images in the delayed phase. In FLAIR images, the CSF and dura mater exhibited similar enhancement, rendering it difficult to differentiate between them and to assess dural enhancement. Table 3 presents the number of cases with contrast enhancement in each region, comparing the CVD affected and unaffected sides. All five large infarction cases (≥ 5 cm) had delayed meningeal enhancement on the ipsilateral side, while none of the remaining six small infarction cases (< 5 cm) exhibited delayed meningeal enhancement on MRI. More than half of the entire cases had delayed white matter enhancement adjacent to the infarction, suggesting much broader involvement than the infarct sites. There was no correlation between the affected side and delayed enhancement of the CNs or venous sinuses.

**Table 3:** Comparison of contrast enhancement in each region on the CVD affected and unaffected sides

Category		Lesion side	Unaffected side
A	Subarachnoid space	5 (45.4%)	0 (0%)
	Dura mater	5 (45.4%)	0 (0%)
	Deep white matter	7 (63.6%)	0 (0%)
	Subdural space	1 (9.1%)	0 (0%)
B	Anterior SSS	11 (100%)	11 (100%)
	Middle SSS	11 (100%)	11 (100%)
	Posterior SSS	11 (100%)	11 (100%)
	Sigmoid sinus	11 (100%)	11 (100%)
	Jugular foramen	11 (100%)	11 (100%)
	Bridging vein	4 (36.4%)	0 (0%)
C	CN I	2 (18.2%)	2 (18.2%)
	CN II	4 (36.4%)	5 (45.5%)
	CN III	3 (27.3%)	3 (27.3%)
	CN V	1 (9.1%)	1 (9.1%)
	CN VII & VIII	6 (54.5%)	7 (63.6%)
D	Anterior chamber	1 (9.1%)	1 (9.1%)
	Pineal gland	8 (72.7%)*	
	Choroid plexus	11 (100%)	11 (100%)
	Eustachian tube	11 (100%)	11 (100%)
	Auricular cartilage	11 (100%)	11 (100%)

Category A: brain parenchyma, juxtacortical subarachnoid space and dura mater; Category B: dural sinus and veins; Category C: cranial nerves; Category D: miscellaneous regions. SSS, superior sagittal sinus, CN, cranial nerves. *The pineal gland is a midline structure

**Fig. 4:**
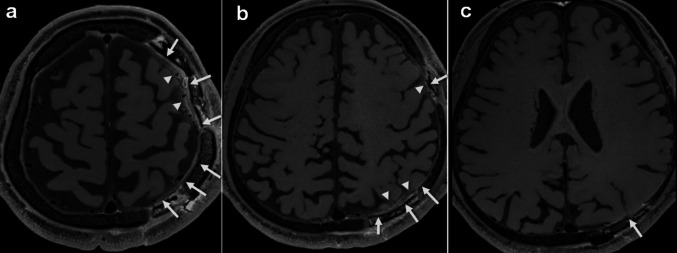
Representative Delayed DANTE T1-SPACE images of a MMD patient (Case 3–MMD, Suzuki stage, right, 3; left, 1) in the postoperative state following left superficial temporal artery to middle cerebral artery (STA–MCA) and left occipital artery to posterior cerebral artery (OA–PCA) bypass surgeries (**a**–**c**). In the postoperative area, delayed enhancement was observed in the dura mater (arrows) and in the subarachnoid space (arrowheads). Except for the postoperative areas, no enhancement was observed in the dura mater, subarachnoid space, and subdural space

Images from representative MMD cases are shown in Fig. 4. In MMD, no case showed delayed enhancement in the subarachnoid space, dura mater, or subdural space on either the ipsilateral or contralateral side, except at the postoperative site (Table 4). In the postoperative area, delayed enhancement was observed in the subarachnoid space in 4 out of 5 cases, and in the dura mater and subdural space in all 5 cases.

**Table 4:** Comparison of contrast enhancement in each region on the MMD affected, unaffected sides, and postoperative area

Category		Affected side (9 sides)	Unaffected side (3 sides)	Postoperative area (5 sides)
A	Subarachnoid space	0 (0%)	0 (0%)	4 (80%)
	Dura mater	0 (0%)	0 (0%)	5 (100%)
	Deep white matter	0 (0%)	0 (0%)	N/A
	Subdural space	0 (0%)	0 (0%)	5 (100%)
B	Anterior SSS	9 (100%)	3 (100%)	N/A
	Middle SSS	9 (100%)	3 (100%)	N/A
	Posterior SSS	9 (100%)	3 (100%)	N/A
	Sigmoid sinus	7 (77.8%)	3 (100%)	N/A
	Jugular foramen	8 (88.9%)	3 (100%)	N/A
	Bridging vein	0 (0%)	0 (0%)	5 (100%)
C	CN I	0 (0%)	0 (0%)	N/A
	CN II	0 (0%)	0 (0%)	N/A
	CN III	0 (0%)	0 (0%)	N/A
	CN V	1 (11.1%)	0 (0%)	N/A
	CN VII & VIII	0 (0%)	0 (0%)	N/A
D	Anterior chamber	0 (0%)	0 (0%)	N/A
	Pineal gland	6 (100%)*		
	Choroid plexus	9 (100%)	3 (100%)	N/A
	Eustachian tube	9 (100%)	3 (100%)	N/A
	Auricular cartilage	9 (100%)	3 (100%)	N/A

Category A: brain parenchyma, juxtacortical subarachnoid space and dura mater; Category B: dural sinus and veins; Category C: cranial nerves; Category D: miscellaneous regions. SSS, superior sagittal sinus; CN, cranial nerves; MMD, moyamoya disease. *The pineal gland is a midline structure

### Interrater reliability

The Fleiss’ kappa values of inter-rater agreement of CVD cohort were 0.75 for Category A, 0.38 for Category B, 0.11 for Category C, and 0.52 for Category D regions. The Fleiss’ kappa values of inter-rater agreement of MMD cohort were 0.87 for Category A, 0.21 for Category B, −0.18 for Category C, and 0.54 for Category D regions.

### Phantom measurements

**Fig. 5:**
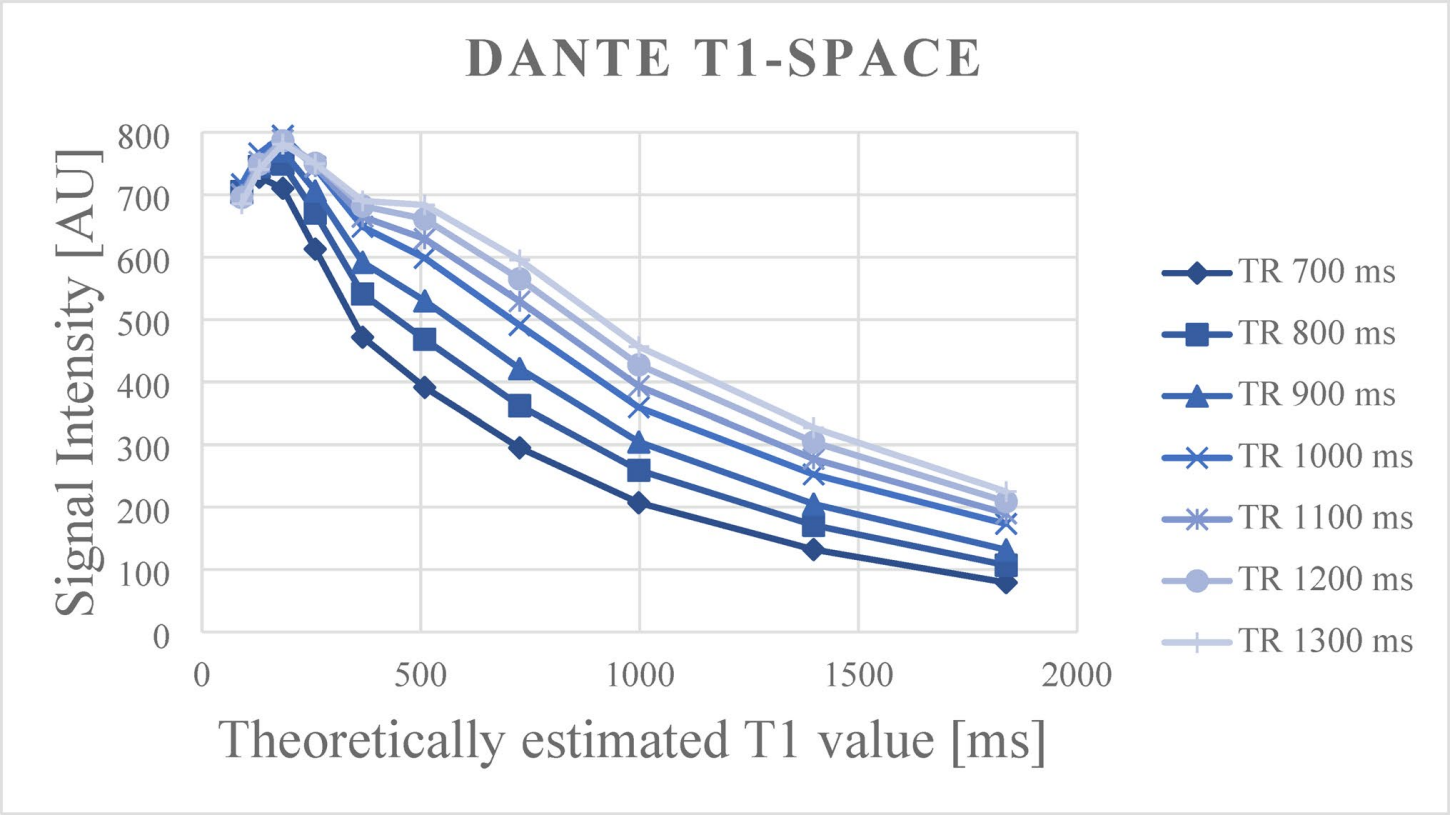
Mean signal intensity on DANTE T1-SPACE of each sphere of the phantom, obtained at various TRs, plotted according to their theoretically estimated T1 values. The signal intensities gradually decreased with increasing T1 values across all TR settings

Figure 5 shows the mean signal intensity on DANTE T1-SPACE of each sphere of the phantom, obtained at various TRs, plotted according to their theoretically estimated T1 values.

The signal intensities gradually decreased with increasing T1 values across all TR settings. In clinical post-contrast images of stroke and MMD patients, regions showing enhancement generally exhibited signal intensities ranging from 200 to 600 [arbitrary unit]. This signal intensity range corresponds approximately to T1 values between 500 and 1000 ms in the phantom results, depending on the TR. Spheres with very short T1 values (< 100 ms), such as T1-11 to T1-14, showed high signal intensities (above 600), likely due to due to central RF interference or B1 inhomogeneity specific to the DANTE T1-SPACE sequence in central of the phantom. These data points were excluded from analysis. Moreover, since such extremely short T1 values rarely exhibit high signal intensity in actual delayed post-contrast images, their exclusion from the analysis was considered appropriate and did not compromise the clinical relevance of our evaluation. These findings indicate that DANTE T1-SPACE is sensitive to intermediate levels of contrast agent retention (T1 value ≈ 500–1000 ms), supporting its suitability for visualizing subtle blood-brain barrier-independent clearance pathways in the delayed phase.

**Fig. 6:**
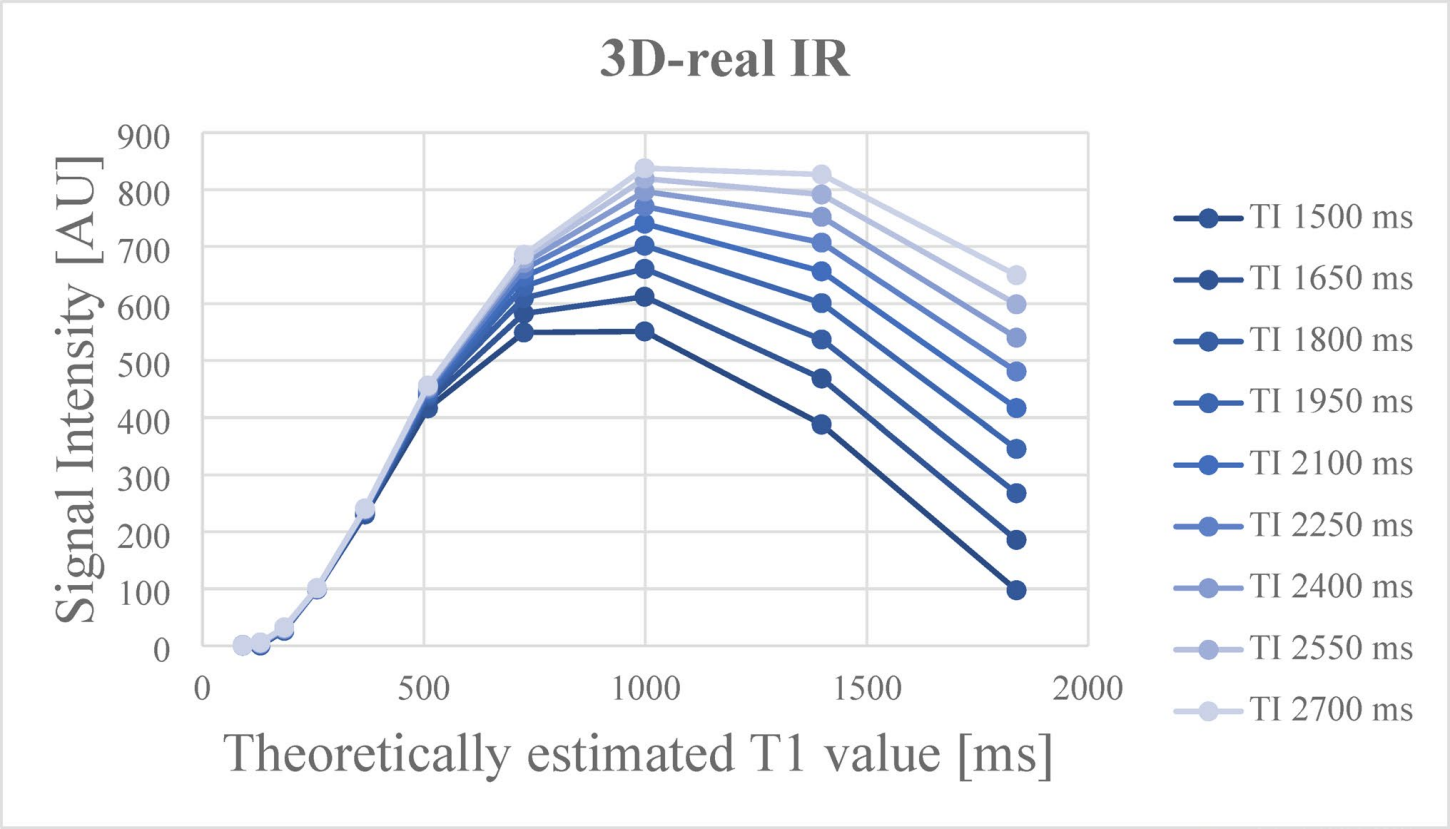
Mean signal intensity on 3D-real IR of each sphere of the phantom, obtained at various TRs, plotted according to their theoretically estimated T1 values. In 3D-real IR, signal intensity increases up to T1 values around 1000 ms, beyond which the signal begins to drop due to the inversion recovery effect. Notably, 3D-real IR demonstrated relatively high sensitivity in the low T1 range

For reference, an additional phantom scan using 3D-real IR is presented in Fig. 6. In 3D-real IR, signal intensity increases up to T1 values around 1000 ms, beyond which the signal begins to drop due to the inversion recovery effect. Notably, 3D-real IR demonstrated relatively high sensitivity in the low T1 range.

## Discussion

In this study, to observe interstitial fluid (ISF) clearance pathways in patients with cerebral infarction, we used CE VW-MRI using DANTE T1-SPACE in the delayed phase. Evaluations showed delayed enhancement of the meninges and dura mater surrounding the infarcted region in cases with relatively large infarct areas. By targeting cerebral infarction, we observed changes in clearance pathways, revealing previously unreported routes through the dura mater beyond the MLVs around the dural sinuses. We believe that delayed enhancement of the meninges and dura mater surrounding the infarcted region indicates the pathway immediately upstream of the MLVs surrounding the dural sinuses and cranial nerves, as previously reported [12, 14, 15, 20–23]. While previous animal experiments on stroke demonstrated impairment of perivascular drainage pathways probably due to decreased arterial pulsation or because of perivascular space occlusion or compression by intravascular thrombus [24], to the best of our knowledge, this is the first study to evaluate the clearance pathway of GBCA in patients with cerebral infarction.

The glymphatic system and intramural periarterial drainage (IPAD) are thought to play essential roles in brain homeostasis through waste clearance [25]. Aspelund et al. [20]. and Louveau et al. [26]. showed that MLVs are downstream pathways of this ISF drainage, after the glymphatic system and IPAD, and before ISF reaches the cervical lymph nodes. Reportedly, MLV dysfunction has been reported in various mouse models of neurological disorders, including multiple sclerosis, Alzheimer’s disease, ischemic stroke, intracerebral hemorrhage, traumatic brain injury, and brain tumors [27]. MLVs have thus become attractive as potential therapeutic targets against CNS pathologies [28–38]. Several previous reports have focused on MR findings of MLV: CE T2-FLAIR and black blood T1-weighted images visualized MLVs along SSS [12]. Naganawa et al. reported that aging significantly accelerates the leakage of GBCA from the subpial space around the cortical veins to the surrounding subarachnoid space, as demonstrated using 3D real inversion recovery (3D-real IR) imaging four hours after intravenous administration of GBCA [37, 39]. A previous animal study showed impairment of the perivascular drainage system or glymphatic system in the acute phase of ischemic stroke [25]. However, while the association between stroke and ISF drainage has gained attention, no study has evaluated the downstream drainage of ISF, including MLVs, in human stroke patients.

MLVs were first reported in rodents, based on observation of fluorescent CSF tracer drainage into the sagittal and transverse dural sinuses [26], and on drainage of dye injected into the brain parenchyma into CSF along perisinus routes and the CSF space before drainage to deep cervical lymph nodes [20, 40]. The above-mentioned pathway is now thought to consist of dorsal MLVs. In later animal studies, lymphatic vessels were reported to be the major outflow pathway for CSF, and ventral MLVs at the skull base [21], including perineural drainage along CN I, CN II, CN V, CN VII, CN IX, CN X, and CN XI, were the hotspots for lymphatic drainage of CSF, but not those in the dorsal perisinus region [21, 22]. However, rodents and the human brain differ in many aspects, including the volume of CSF and the clearance systems in the brain. For example, clearance of tracer from CSF occurs over a time scale of days in humans [23], compared to a few hours in rats [41]. While nasal lymphatic efflux is consistently shown to be of major importance in rodents [42], an in vivo CSF tracer or intravenously injected GBCA study in humans detected no efflux through the cribriform plate to the nasal mucosa [14, 15]. On human MRI, visualization of perisinus MLVs extending to the cervical LNs were reported with high-resolution T2–FLAIR [23] and T1-weighted black-blood imaging [12]. Perivenous gadolinium leakage was also reported with 3D-real IR and FLAIR imaging [37, 43].

In all cases in our study, delayed enhancement was observed around the SSS, sigmoid sinus, and jugular foramen, consistent with the previously reported visualization of dorsal MLVs [12, 21, 22]. Regarding the CNs, delayed enhancement around CN II and CN VII/VIII was observed in approximately half to more than half of the cases. However, no clear delayed enhancement was noted around the other CNs, including CN I, through the cribriform plate, which is considered a major drainage pathway in rodents [42]. The less distinct drainage pathway around CN I observed in our study is consistent with previous reports in humans [14, 15], suggesting that regions surrounding CN II and CN VII/VIII might play a more prominent role in ISF drainage. However, the observed enhancement in these areas was minimal, with only a small volume enhanced compared to the regions around the dural sinuses. Therefore, it is reasonable to infer that dorsal MLVs are the dominant drainage pathway in humans. Additionally, since the delayed enhancement in these areas showed no laterality between the affected and unaffected sides, we assumed that some upstream compensatory mechanisms might alleviate the effects of cerebral infarction, leading to the absence of laterality when these downstream pathways are reached.

Phantom validation also demonstrated that the DANTE T1-SPACE sequence (TR = 1000 ms) exhibited good sensitivity to T1 values in the range of approximately 500–1000 ms. This range corresponds to signal intensities (200–600 arbitrary units) typically observed in regions showing delayed enhancement in clinical post-contrast images. These findings support that the imaging parameters used in this study were adequate for detecting subtle gadolinium retention relevant to clinical evaluation. Compared to 3D-real IR, which shows increasing signal intensity up to T1 values around 1000 ms followed by a decline due to the inversion recovery effect, DANTE T1-SPACE demonstrated more stable and monotonic signal attenuation across T1 values above 200 ms. This signal behavior in DANTE T1-SPACE corresponds to the intensity range typically seen in clinical delayed-phase enhancement (signal intensities of 200–600), suggesting that this sequence may be useful not only for detecting enhancement but also for visually assessing its relative degree in a clinically meaningful T1 range.

We also observed contrast enhancement in the dura mater and CSF space in this study, which has not been previously reported in delayed CE MRI studies. All cases showing dural enhancement were associated with large infarct areas, with the enhancement being most prominent in regions where the infarct extended to the cortical surface. Although residual GBCA in the CSF space may increase the signal intensity, the enhancement of the CSF space corresponded to the infarcted area. The distinct margins of the enhanced regions rule out the possibility of breakdown of the BBB as a possible cause of the enhancement, instead suggesting possible drainage via the arachnoid mater. Regarding the observed dural enhancement, since the dura mater is unlikely to be affected by BBB disruption due to impairment of the internal carotid artery territory, the dural enhancement observed in this study cannot be fully accounted for by the MLVs previously identified around the venous sinuses [12, 14, 15, 20–23]. It is, therefore, plausible that compensatory excretory routes toward the dura mater are activated in response to impairment of the usual drainage pathways, such as IPAD and the glymphatic system, following cerebral infarction.

To further explore the specificity of these findings, we included a cohort of patients with MMD, in whom extensive collateral circulation is typically present. Notably, no delayed enhancement was observed in the dura mater or CSF spaces outside the postoperative regions in these patients. This comparison supports the interpretation that the delayed enhancement observed in cerebral infarction likely reflects altered interstitial fluid clearance, rather than collateral circulation or inflammation.

There are several limitations to this study. First, we were unable to maintain consistent elapsed time after GBCA injection and were also unable to perform dynamic studies due to the limited availability of time slots for MR imaging. However, since delayed contrast enhancement has been observed between 1.5 and 6 h after contrast administration in previous studies [39, 44], this was unlikely to have influenced the presence of enhancement. In regions like CNs and around dural sinuses, where enhancement is less pronounced, variability could be related to the differences of elapsed time after GBCA injection or individual differences. Second, the sample size in this study was small. Further research with larger sample sizes and dynamic imaging studies are necessary to better understand the impact of cerebral infarction on glymphatic and meningeal lymphatic pathways. Third, it is unclear whether the areas with contrast enhancement truly represent glymphatic system activity. It is also possible that such enhancement is influenced by post-stroke meningeal immune responses, including mast cell–mediated recruitment of immune cells and cytokine release [45, 46], as well as reactive changes in MLVs. However, in our cohort, the absence of enhancement in the immediate post-contrast phase and its appearance only in the delayed phase suggest that these findings are more consistent with an altered GBCA clearance route through the MLV network, rather than enhancement primarily driven by immune cell infiltration, inflammation, or simple leakage.

In conclusion, our study observed delayed enhancement of the dura mater, pia mater, and CSF around large infarctions in patients with cerebral infarction. These findings could represent upstream pathways of MLVs around the dural sinuses. The MMD cohort strengthens the interpretation that the delayed enhancement likely represents alternative drainage pathways rather than collateral vasculature.

## Data Availability

The datasets used and/or analyzed during the current study are available from the corresponding author on reasonable request.
